# Association Between Abdominal Waist Circumference and Blood Pressure In Brazilian Adolescents With Normal Body Mass Index: Waist circumference and blood pressure in Adolescents

**DOI:** 10.5334/gh.779

**Published:** 2020-04-03

**Authors:** Daiane Cristina Pazin, Tatiana Lorena da Luz Kaestner, Márcia Olandoski, Cristina Pellegrino Baena, Gabriela de Azevedo Abreu, Maria Cristina Caetano Kuschnir, Katia Vergetti Bloch, José Rocha Faria-Neto

**Affiliations:** 1 School of Medicine, Pontifícia Universidade Católica do Paraná, Rua Imaculada Conceição, Curitiba/PR, BR; 2 Center for Clinical and Epidemiological Research (EpiCenter), School of Medicine, Pontifícia Universidade Católica do Paraná, Rua Rockfeller, Curitiba/PR, BR; 3 Institute of Public Health Studies, Federal University of Rio de Janeiro, Avenida Horácio Macedo S/N, Rio de Janeiro, RJ, BR; 4 School of Medicine. Rio de Janeiro State University, Rio de Janeiro, RJ, BR

**Keywords:** body mass index, waist circumference, blood pressure, cross-sectional studies, public health, adolescent

## Abstract

Obesity is the most common chronic disease in adolescents. In adults, waist circumference (WC) is associated with the presence of cardiovascular risk factors and is also a better predictor of cardiovascular (CV) risk than body mass index (BMI). The association between WC and CV risk factors in adolescents has been poorly explored so far, mainly in those within the normal BMI range.

**Objective:**

To evaluate the association between WC and elevated blood pressure (BP) in adolescents with a normal BMI.

**Methods:**

Cross-sectional analysis of 73,399 scholars between 12 and 17 years old from the ERICA study, a school-based, national representative study with Brazilian adolescents. Only those within the normal range of BMI were included. The WC was categorized into quartiles for sex and age (Q1 to Q4). For the analysis, BP values ≥ 90th percentile were considered to indicate elevated BP, what includes hypertension and pre-hypertension. The Poisson Regression model was used and the prevalence ratio was estimated.

**Results:**

A total of 53,308 adolescents with normal BMI were included. Prevalence of elevated BP in the overall group was 18.0%. In female adolescents with WC in the lowest quartile for their age, the prevalence of elevated BP was 7.3% (12–14 years) and 6.9% (15–17 years), increasing in the upper quartile to 15.2% and 19.5% respectively, with a prevalence ratio (PR) indicating chance at least two times higher for elevated BP in Q4 (p < 0.001). Similarly, this was observed in boys, with a prevalence of elevated BP of 10.0% and 18.9% in Q1, increasing to 21.4% and 49.6% in Q4 (p < 0.001).

**Conclusion:**

In adolescents, there is a strong association of increased WC with BP elevation, even when the BMI is adequate.

## Introduction

Cardiovascular diseases (CVDs) are the leading cause of death worldwide. CVD mortality rates are influenced by several risk factors, such as smoking, changes in cholesterol and glucose levels, and also treatment availability and quality [1]. Hypertension is one of the most challenging risk factors in public health [2]. Although hypertension is more prevalent in the elderly, there has been an increase in the prevalence of hypertension in adolescents worldwide. During adolescence, hypertension is usually essential, not secondary, and influenced by the excess of weight, metabolic syndrome (MetS), and genetic factors [3].

Adolescence is a stage between the ages of 12 to 18 years, during which a series of psychological, hormonal, and anthropometric changes occur. Obesity is the most common chronic disease in this age group [4] and is related to a future decrease in life expectancy [5]. Among several cardiovascular (CV) risk factors evaluated in children and adolescents, overweight was associated with a higher prevalence of high cholesterol levels, diastolic blood pressure (BP), triglyceride level, and fasting glucose level [6,7]. Body mass index (BMI) is the most widely method used for assessing overweight. However, it is mainly the excess of body fat that is related to the risk of chronic diseases, not being overweight [8]. BMI does not provide information on abdominal fat, which is metabolically more active and is well defined as an independent risk factor for CVDs in adults [9]. On the other hand, waist circumference (WC) is a better marker of abdominal fat and has a stronger association with CV risk factors [10].

In adolescents, the use of WC is limited due to changes in body composition caused by growth and development [10]. WC increase is not necessarily associated with BMI increase, and can be abnormal even when it is within the normal range [11]. In addition, many CV risk factors are present in children and adolescents with normal weight [12]. Therefore, measures such as neck circumference, WC, hip circumference, and waist-to-hip ratio, can be used to predict arterial hypertension, glucose metabolism, others CVD changes, and thus help identify those who need more attention [13].

The Study of Cardiovascular Risk in Adolescents – ERICA was the first nationally represented Brazilian study, designed to provide estimates of the prevalence of diabetes mellitus, obesity, CV risk factors, insulin resistance, and inflammatory markers in adolescents [14,15,16,17]. The present study aims to evaluate the association between WC and elevated BP in adolescents aged 12 to 17 years with a normal BMI.

## Methods

## Study design and sample

The data analyzed were obtained from ERICA, a cross-national, school-based study. It is one of the most comprehensive studies on the prevalence of CV risk factors in adolescents ever conducted in Brazil and was approved by the Research Ethics Committees of the study’s central coordinating institution (IESC/UFRJ) and of each Brazilian state.

The sample was divided into 32 strata consisting of the 27 Brazilian capital cities and five sets of countryside cities containing more than 100,000 inhabitants from each of the country’s five macro-regions. Thus, the sample is representative nationally, regionally and for the capitals and the Federal District. Schools were sampled using probability proportional to size (PPS), with the measure of the size equal to the ratio between the number of the students of the school, and the distance, in kilometers, between the municipality where the school was located and the capital of the state, to avoid the spread of the sample. The systematic PPS selection was used, with schools sorted by geographic stratum, location (urban x rural) and administrative dependency (public or private). In the second stage of sampling, three combinations of shift and grade were sampled. All eligible students of the sampled classes were included. The sampling process has been described in prior publications [18,19].

The observers were trained by ERICA’s central coordination team, according to the study protocol. An operation manual and videos were especially produced for the training of anthropometric and BP measures. The observers were trained in anthropometric measurements using the Habicht’s criterion [20] as a guide to assess inter and intra variability. Checks were conducted regularly to identify outliers, discrepancies or digit preference in measurements [18].

Between March 2013 and December 2014, a pre-trained team evaluated adolescents aged 12 to 17 years attending 1,248 schools in 121 Brazilian municipalities. An anthropometric and BP evaluation was performed; furthermore, 24-hour food abstinence was instructed, and blood laboratory tests were performed. A self-filled questionnaire with items on socio-demographic and lifestyle information was also employed.

## Exclusion criteria

The study used data from adolescents of both sexes with a normal BMI [21]. We excluded from the analysis: pregnant adolescents; those with temporary or permanent physical disabilities; those who refused to participate in the study; those who did not sign the informed consent form (ICF) or did not bring the ICF signed by the individual responsible, when required by the municipality.

## Ethical issues

This study was conducted according to the principles of the Helsinki declaration. The approval of the Ethical Committee at each of the 26 States and for the Federal District was obtained. Permission to conduct the study was obtained in all State and local Departments of Education and in all schools. Written informed consent was obtained from each student after carefully explanation about the study and its procedures, and also from their parents for those who are invited to take blood collection (those studying in the morning). The student’s privacy and confidentiality were guaranteed throughout the study.

## Data collection

Weight was evaluated using the Leader model P200M electronic scale with a capacity of up to 200 kg and variation of 50 g. Stature was measured twice using the Exact Height portable stadiometer with a variation of 0.1 cm, considering the average of the two values obtained. Weight and height were used to classify the nutritional status from the BMI calculation (weight/height); the specific BMI curves proposed by the World Health Organization (2007) for age and sex were employed. The cut-off point used was as follows: Z score of –2 to 1 (adequate weight) [22].

WC was measured using a Sanny inextensible measuring tape with a variation of 0.1 cm at the midpoint between the lower curvature of the last fixed rib and the superior curvature of the iliac crest, with the adolescents standing and with their arms alongside the body, feet together, and abdomen relaxed [17]. The WC was categorized into quartiles for sex and age in adolescents with a normal BMI.

BP was measured and classified according to the Fourth Diagnosis, Evaluation [23], and High Blood Pressure Treatment in Children and Adolescents, which considers sex, height, and age. A digital monitor (Omron 705-IT) validated for use in adolescents was used with a cuff appropriate for the arm perimeter of the right arm. The students were seated with their feet flat on the floor. Three measurements were obtained at 3-minute intervals, using the average of the last two measurements. According to the proposed classification, adolescents are considered normotensive when BP is below 90 percentile; pre-hypertensive (normal-elevated BP) when BP is between the 90th and the 95th percentiles; and hypertensive when BP is equal or above 95th percentile. In the present study, BP values ≥ 90th percentile were defined as ‘elevated BP’.

## Statistical analysis

For each sex and age (12 to 17 years), restricted to adolescents with adequate nutritional status, the quartiles of the WC measures were calculated. Thereafter, the adolescents were classified into one of the quartiles according to their age and sex. Age was analyzed in two aged - groups, 12–14 years and 15–17 years, due to the homogeneity in anthropometric and blood pressure parameters in each age group.

The association between WC and BP, considering two BP levels (normal and elevated), was analyzed by adjusting the Poisson Regression model, estimating the prevalence ratio (PR) and the respective 95% confidence intervals. All analyses were performed considering the complex sampling design. Data were analyzed using the Stata/SE v.14.1 survey module (StataCorp LP, US). In addition to the natural weights of the sample design, post-stratification estimators that modified natural weights by calibration factors were used. Those calibration factors were obtained by dividing population totals by totals estimated by natural weights for the post-strata, defined as 12 estimation domains, corresponding to age and sex combinations, considering the population data of adolescents enrolled in all schools). P values of < 0.05 were considered to indicate statistical significance.

## Results

### Sample characteristics

Complete data on anthropometry and BP were obtained from 73,399 students out of a total of 102,327 eligible individuals between the ages of 12 and 17 years, of whom 53,308 had a normal BMI. A total of 2,159 (2.94%) students were excluded for being underweight and 17,932 (24.4%) for presenting excess weight (overweight: 12,292, 16.8%; obesity: 5,640, 7.7%). The mean age was 14.9 years, and 29,995 (56.3%) were girls; further, approximately 84% were public school students, reflecting the source population distribution.

The estimated prevalence of elevated BP in the sample was 18.0% (95%CI 17.0–19.2), of these, pre-hypertensive were 11.7% (95%CI 11.0–12.5%) and hypertensive 6.3% (95%CI 5.7–7.1%). The distribution of BMI, WC, and the prevalence of elevated BP by sex and age are presented in Table 1. The means of BMI and the prevalence of elevated BP increase with age in both sex and males have higher WC means and elevated BP prevalence.

**Table 1:** Means of body mass index and waist circumference and prevalence of elevated blood pressure and 95% confidence intervals in adolescents with adequate BMI.

Sex	Age group (years)	n	BMI Mean (95%CI)	WC Mean (95%CI)	Elevated BP% (95%CI)

Female					
	12–14	18,497	19.0 (18.9–19.1)	65.4 (65.2–67.7)	10.1 (9.0–11.3)
	15–17	22,178	20.3 (20.1–20.4)	68.4 (68.2–68.7)	12.7 (11.1–14.6)
	All	40,675	19.6 (19.6–19.7)	66.9 (66.8–67.1)	11.4 (10.3–12.6)
Male					
	12–14	15,178	18.3 (18.2–18.4)	66.0 (65.8–66.3)	15.2 (13.9–16.8)
	15–17	17,546	20.2 (20.1–20.2)	71.0 (70.9–71.4)	34.8 (32.6–37.1)
	All	32,724	19.2 (19.2–19.3)	68.5 (68.3–68.7)	24.8 (23.3–26.4)

BMI, Body Mass Index; WC, Waist Circumference; BP, Blood Pressure; CI, Confidence Interval.

### Association between WC and BP

Among the students with an adequate BMI, the association between WC and BP was dependent on age and sex. Considering the first quartile as a reference for both, boys and girls, the prevalence of elevated BP was higher in the largest WC quartiles for each defined stratum. For the adolescent girls aged between 12 and 14 years, the prevalence of elevated BP in Q4 of WC was more than two times higher than that in Q1. Moreover, the prevalence was almost three times higher among those aged between 15 and 17 years. Similarly, the boys aged 12 to 14 years in Q4 had a twice higher prevalence of elevated BP, whereas those aged 15 to 17 years had a 2.5 times higher prevalence (Table 2).

**Table 2:** Elevated blood pressure prevalence according to quartile of Waist Circumference by sex and age – group.

Sex	Age group (years)	Waist circumference Cut-off point (cm)	Prevalence of elevated BP (95% CI)	p-value*	PR (95%CI)*

Female		1st quartile (<61.9) (ref)	7.3 (5.5–9.6)		–
	12 to 14	2nd quartile (≥61.9; <65.2)	8.4 (6.4–10.9)	0.520	1.15 (0.75–1.76)
		3rd quartile (≥65.2; <68.9)	9.5 (7.8–11.5)	0.137	1.30 (0.92–1.82)
		4th quartile (≥68.9)	15.2 (11.8–19.3)	0.001	2.07 (1.33–3.23)
	15 to 17	1st quartile (<64.6) (ref)	6.9 (5.7–8.4)		–
		2nd quartile (≥64.6; <68.0)	11.8 (8.7–15.8)	0.003	1.71 (1.20–2.44)
		3rd quartile (≥68.0; <71.8)	12.6 (10.0–15.7)	<0.001	1.83 (1.38–2.43)
		4th quartile (≥71.8)	19.5 (15.8–23.9)	<0.001	2.83 (2.20–3.65)
Male		1st quartile (<62.8) (ref)	10.0 (7.8–12.7)		–
	12 to 14	2nd quartile (≥62.8; <66.2)	14.8 (9.8–21.7)	0.142	1.49 (0.88–2.53)
		3rd quartile (≥66.2; <69.5)	14.6 (12.4–17.2)	0.021	1.46 (1.06–2.03)
		4th quartile (≥69.5)	21.4 (18.4–24.8)	<0.001	2.15 (1.61–2.86)
	15 to 17	1st quartile (<67.7) (ref)	18.9 (15.9–22.2)		–
		2nd quartile (≥67.7; <70.6)	29.3 (25.2–33.9)	<0.001	1.56 (1.25–1.93)
		3rd quartile (≥70.6; <74.0)	41.4 (37.6–45.3)	<0.001	2.19 (1.81–2.66)
		4th quartile (≥74.0)	49.6 (46.5–52.8)	<0.001	2.63 (2.18–3.17)

BP, blood pressure; PR, prevalence ratio; CI, confidence interval.

It is possible to observe an increasing tendency into quartiles for both sexes in all defined age strata. Although the association between WC and BP is stronger for the older boys, there is also a significant gradient observed for girls.

## Discussion

The correlation between excess of weight, evaluated by BMI, and elevation of blood pressure is well known, but the role of other anthropometric measures to identify individuals at risk when BMI is normal has been poorly explored so far. In this study, we demonstrated in a large population of adolescents between 12 and 17 years old within the normal range of BMI that increased WC is associated with a higher prevalence of elevated BP. This is a novel finding with clinical implication for the screening of elevated blood pressure among adolescents, especially considering the growing prevalence of hypertension worldwide [24].

The prevalence of high blood pressure among adolescents is increasing [3]. In our study, the prevalence of elevated BP was compatible with recent reports which grouped pre-hypertensive and hypertensive status in the analysis [25,26]; and also with the total population of ERICA study, in which 14.5% pre-hypertensive and 9.6% hypertensive adolescents were identified, aiming total prevalence of elevated BP of 24.1% [14]. Despite that, it is a not common practice to screen for hypertension among children and adolescents routinely [27]. Many countries and organizations have established their own BP reference values [28], without a consensus, making hypertension identification even more difficult. In fact, recommendation of screening for hypertension is insufficient [29], and is currently under review. At the present, only the evaluation of BP in individuals above the age of 18 years is recommended [30]. Our results reinforce the need for screening, indicating that WC is a valuable marker to help in the identification of those at risk for hypertension, even when BMI is normal.

BMI and WC are both indicators of adiposity that correlate with hypertension in the general population [27,31,32]. In adults, WC is a reliable marker of abdominal fat as demonstrated by imaging methods and is an independent and well-established risk factor for CVDs [33]. WC is also considered a predictor of visceral adiposity and hypertension in the young population [27,31]. Data describing noninvasive biomarkers for use in screening of adolescents and young adults are limited [13]. When comparing anthropometric measurements and abdominal fat assessment using imaging methods for children and adolescents, WC shows a better correlation with visceral adiposity then other anthropometric parameters, although only a few studies have proposed this analysis [34]. In obese Mexican adolescents, the waist-to-hip ratio was superior in identifying cardio-metabolic risk factors [35] but in larger samples, BMI and WC performed better [27,36]. Recently, neck and wrist circumferences have emerged as useful measures in demonstrating the central distribution of fat; however, they could only serve as an alternative, as the predictive potential is still undetermined [37,38]. There is a criticism regarding WC in that it does not allow the quantification of adipose tissues, nor does it distinguish visceral fat from subcutaneous fat. Also, there is no standardized method for measuring WC or defined reference values for children and adolescents [37,39].

Although our study has the strength of a very large sample, it has also some limitations. Similar to several studies that analyzed anthropometric measures and associated factors in children and adolescents, this is a cross-sectional study that does not allow clinical diagnosis of hypertension to be established. Also, BP levels were obtained at a single visit, which may overestimate elevated BP prevalence. However, because of the young age of the study population it is unlikely that the association observed is due to reverse causality or survival bias, two important problems related to inference in cross-sectional studies. The categorization of the waist perimeter per quartile minimizes measurement variation problems, since no cutoff values are defined for this population. Besides that, we were able to evaluate the WC independent of BMI.

## Conclusion

We have demonstrated that even in adolescents with a normal BMI, increased WC is strongly associated with elevated BP. Our findings reinforce the need of assessment of blood pressure in adolescents, and evaluation of obesity in this population shall not be restricted to BMI assessment, but also WC. Moreover, definition of cut-off points for WC in adolescents would facilitate its use in clinical practice.

## Abbreviations

Cardiovascular disease – CVD

Metabolic syndrome – MetS

Cardiovascular – CV

Blood pressure – BP

Body mass index – BMI

Waist circumference – WC

Probability proportional to Size – PPS

Informed consent form – ICF

Prevalence ratio – PR

## Author Information

  **Daiane Cristina Pazin**
  Family phisycian graduated from the Pontifical Catholic University of Paraná (PUCPR) (2007), specialization (2008-2010) and a master’s degree in Health Sciences (2016). Professor at PUCPR School of Medicine, working with the board of Family and Community Medicine, and at Positivo University School of Medicine.
  **Tatiana Lorena da Luz Kaestner**
  Professor of Family and Community Medicine at PUCPR. Current coordinator of the Family and Community Medicine residency at Cajuru University Hospital and teacher responsible for the academic league of Primary Care. Graduated in 2007 from PUCPR. Specialist in Family and Community Medicine (2009). Master’s degree in Health Sciences (2016).
  **Márcia Olandoski**
  Graduated in Mathematics at the Federal University of Paraná (1976), Master in Statistics at the Federal University of Rio de Janeiro (UFRJ) (1981) and PhD in Health Sciences at the PUCPR (2010). Currently Professor of Biostatistics at PUCPR School of Medicine. Has been working with Biostatistics for 23 years, working on topics such as acute myocardial infarction, presyncope, syncope, mesenchymal stem cells, celiac disease, heart failure, kidney disease, orthodontics, among others, but with flexibility to work with any subject in the area of health.
  **Cristina Pellegrino Baena**
  Degree in Physiotherapy at PUCPR (1996). Master’s degree in Health Sciences at Federal University of Rio Grande (2010) and a Doctorate in Health Sciences at PUCPR in 2013. Sandwich grant from Capes to research with the Cardiovascular Epidemiology group of Erasmus University in Rotterdam/Netherlands (2012). Postdoctoral researcher at the Epidemiological Research Center of the University Hospital of the University of São Paulo in cardiovascular epidemiology projects. Today, professor at School of Medicine/PUCPR and currently coordinator of the Graduate in Health Sciences (Master and Doctorate) at PUCPR.
  **Gabriela A Abreu**
  Degree in Nutrition from the Federal University of the State of Rio de Janeiro (UNIRIO), Specialization in Clinical Nutrition from the Federal University of Rio de Janeiro (UFRJ), Master and Doctorate in Collective Health by the Institute of Collective Health Studies (IESC) of UFRJ and Post-Doctorate in Collective Health by the Institute of Social Medicine, Rio de Janeiro State University (IMS/UERJ).
  **Maria Cristina Caetano Kuschnir**
  Degree in Medicine from the State University of Rio de Janeiro (UERJ) (1982), Medical Residency in Pediatrics with expertise in Adolescent Medicine. Master’s Degree in Women’s and Children’s Health from the Oswaldo Cruz Foundation (1996) and PhD Epidemiology - Collective Health (2004). Currently Adjunct Professor of Adolescent Medicine - UERJ, Professor of Master Courses and Doctorate of the Postgraduate Program in Medical Sciences. Part of the Central Coordination of the Study of Cardiovascular Risk in Adolescents - ERICA.
  **Katia Vergetti Bloch**
  Associate Professor of Epidemiology and Adjunct Director of Postgraduate Studies and Research at the Institute of Studies in Public Health at Federal University of Rio de Janeiro (UFRJ). Graduated in Medicine from the UFRJ (1983), Masters in Medical Clinic (1990) and PhD in Public Health from the Federal University of Bahia/London School of Hygiene and Tropical Medicine (1997). Coordinated the national execution of the Study of Cardiovascular Risks in Adolescents - ERICA.
  **José Rocha Faria-Neto**
  Graduated in Medicine from the Federal University of Paraná (1994). He held a residency in Clinical Medicine (1995) and Cardiology (1996/97). PhD in Cardiology from the Faculty of Medicine of USP (1998-2002). Postdoctoral fellow at the Atherosclerosis Research Center at Cedars-Sinai Medical Center in Los Angeles (2002-04). Professor at PUCPR of the discipline of Cardiology and the Graduate Program in Health Sciences (CAPES 5).
